# Mesenchymal Stem Cell Therapy for Hutchinson–Gilford Progeria: Improvements in Arterial Stiffness and Bone Mineral Density in a Single Case

**DOI:** 10.3390/children12040523

**Published:** 2025-04-18

**Authors:** Eun-Young Joo, Ji-Sun Park, Hyun-Tae Shin, Myungji Yoo, Su-Jin Kim, Ji-Eun Lee, Gwang-Seong Choi

**Affiliations:** 1 Department of Pediatrics, Inha University College of Medicine, Incheon 22212, Republic of Korea; freegana@inha.ac.kr (E.-Y.J.); pedendo@inha.ac.kr (J.-S.P.);; 2 Gyeonggi-Incheon Regional Specialized Rare Disease Institute, Inha University Hospital, Incheon 22332, Republic of Korea; 3 Advanced Regenerative Medicine Clinical Trial Center, Inha University Hospital, Incheon 22332, Republic of Korea; 4 Department of Dermatology, Inha University College of Medicine, Incheon 22212, Republic of Korea

**Keywords:** progeria, mesenchymal stem cell therapy, arterial stiffness, bone mineral density, atherosclerosis

## Abstract

Background/Objectives: Hutchinson–Gilford progeria syndrome (HGPS) is a rare genetic disorder that cause premature aging due to LMNA mutations and progerin accumulation. Although lonafarnib, an FDA-approved farnesyltransferase inhibitor, offers modest extension of life, the disease remains progressive. As progeria is associated with stem cell depletion and mesenchymal stem cell (MSC) therapy has shown efficacy in treating atherosclerosis, we aimed to evaluate its efficacy and safety in HGPS. Methods: A 7-year-old male with classic HGPS and preexisting severe cerebrovascular disease received four intravenous infusion of bone marrow-derived MSCs (2.5 × 10⁵ cells/kg) over 8 months. Growth, metabolic, cardiovascular, musculoskeletal, auditory, and inflammatory cytokines were monitored throughout the study. Prophylactic enoxaparin was administered to prevent vascular complications. Results: MSC therapy was associated with improved lean body mass (11.5%), bone mineral density (L-spine z-score: 0.55 → 2.03), reduced arterial stiffness (9.98% reductionin pulse wave velocity), joint range of motion, dentition, and decreased sICAM-1 levels. However, Cardiovascular deterioration continued, and the patient passed away 10 months after the fourth dose, likely due to progression of the underlying vascular disease. No severe adverse effects were attributed to MSC therapy. Conclusions: MSC therapy may offer short-term benefits in arterial stiffness, bone health and inflammation in HGPS without notable safety concerns. Further studies are warranted to validate these findings, explore earlier intervention, and determine long-term efficacy and optimal dosing strategies.

## 1. Introduction

Classic Hutchinson–Gilford progeria syndrome (HGPS) is a rare premature aging disorder that occurs in approximately 1 in 4 million individuals. It is caused by a mutation in the *LMNA* gene on Chr.1q22 (c.1824C>T, p.G608G) [1,2], leading to progerin accumulation. Progerin disrupts nuclear envelope stability, impairs DNA repair, and accelerates aging. Progeria patients look similar to normal babies at birth. However, severe growth failure and sclerotic skin changes begin in infancy. Over time, distinctive features, including hair loss, prominent eyes, micrognathia, a beaked nose, lipodystrophy, and joint stiffness, become evident [3,4,5]. After the age of 2, the mean growth rate is only 3.58 cm/year, and weight gain is limited to 0.44 kg/year [6,7]. Progerin promotes rapid atherosclerosis through vascular smooth muscle cell depletion and endothelial dysfunction, leading to the formation of unstable plaques [8,9]. The mean life expectancy is about 14.5 years, with cardiovascular complications being the primary cause of death [10,11].

Lonafarnib (Zokinvy^®^), a farnesyltransferase inhibitor, is the only FDA approved drug for progeria, extending life expectancy by an average of 2.5 years [12,13]. It also provide positive effects on weight gain, cardiovascular stiffness, bone density, and hearing [14]. However, high cost, frequent gastrointestinal side effects, and limited accessibility in some countries pose barriers to its use. Morever, lonafarnib alone does not halt disease progression, leading to studies on combination therapies, such as zoledronic acid, pravastatin, or everolimus, to improve its efficacy [15,16].

Stem cell exhaustion is characteristic of HGPS. Increased nuclear fragility, increased mitochondrial oxidative stress, and chronic inflammation accelerate high cell turnover, telomere shortening, leading to depletes progenitor cells. [17,18,19]. Depletion of endothelial progenitor cell loss may exacerbate atherosclerosis [19] Progerin further impairs mesenchymal stem cell (MSC) function, contributing to premature aging features [20]. Stem cell depletion is also linked to key clinical signs, such as lipodystrophy, hair loss, joint stiffness, and delayed dentition [19].

Owing to their immunomodulatory, regenerative effects, MSCs therapy hasshown therapeutic potential in treating aging [21,22], atherosclerosis [23,24], and stroke [25] through paracrine and regenerative mechanisms. Commercially available MSC products enhance clinical applicability. Given their potential to address the multifaceted pathology of HGPS, we investigated the efficacy and safety of intravenous (IV) allogeneic bone marrow-derived clonal MSC therapy in a patient with classic r HGPS.

## 2. Case Report

### 2.1. Case Description

The patient was a male aged 7 years and 9 months with classic HGPS at the start of the study. This study protocol was approved by the Korean Ministry of Food and Drug Safety, Advanced Regenerative Medicine (R-3-0005), and informed consent was obtained from the patient and his parents.

The patient was born full term with a birth weight of 2.8 kg (11.8 percentile). However, severe growth failure due to poor feeding and abdominal sclerodermoid changes were observed within one month of birth. By 5 months of age, his weight (5.3 kg) and height (61 cm) had fallen below the 1st percentile. At age 5, the patient was diagnosed with progeria due to an *LMNA* mutation (c.1824C>T). A comprehensive evaluation was conducted at the time of diagnosis. He exhibited hair loss with prominent scalp veins, and impaired nail growth, including joint contractures, particularly in the hand and foot. Brain magnetic resonance angiography (MRA) revealed mild to moderate atherosclerosis in the bilateral internal carotid arteries and severe segmental stenosis of the bilateral vertebral arteries with collateral vessels, although the patient had no preceding neurological symptoms. Atorvastatin and clopidogrel were started on the basis of the vascular state. Echocardiography revealed normal ventricular function with trivial tricuspid regurgitation and pulmonary regurgitation. At age 6 years and 9 months, the patient experienced his first transient ischemic attack (TIA), with symptoms of severe headache, dizziness, and lower extremity weakness, all of which resolved spontaneously.

### 2.2. Study Design

The aim of this study was to evaluate the efficacy and safety of allogenic bone marrow-derived clonal MSCs in a patient with HGPS. This study was designed after patients visited our hospital. The study was planned for 24 months and included screening, treatment, interim evaluation, and follow-up. Five IV administrations of MSCs (2.5 × 10⁵ cells/kg each, SCM-AGH, SCM Life Sciences) were planned. The first three doses were administered at one-month intervals. An interim evaluation was conducted at three months following the third injection, after which two additional doses were administered at six-month intervals. 

The inclusion criteria were as follows: (1) a typical phenotype of HGPS, (2) confirmation of HGPS by genetic testing and (3) AST/ALT < 5 times the upper limit of the normal range for age. The exclusion criteria included: (1) severe renal impairment (glomerular filtration rate [GFR] < 30 mL/min/1.73 m^2^), (2) received any other clinical trial treatment 90 days prior to the start of this study, and (3) severe adverse drug reactions during stem cell therapy. 

Comprehensive baseline assessments were performed, including growth (anthropometric, IGF-1, IGFBP3, and age adjusted z -score), metabolic (AST, ALT, insulin, c-peptide, Hba1c, and cholesterol), proinflammatory and atherosclerosis-associated cytokines (TNF-α, IL-1β, IL-8, and IL-18; monocyte chemoattractant protein-1 [MCP-1], and soluble intercellular adhesion molecule-1 [sICAM-1]), brain imaging (MRA), cardiac (chest X-ray, electrocardiography, echocardiography, brachial-ankle pulse wave velocity [baPWV] testing, carotid intima-media thickness [cIMT] measurement), ophthalmologic and auditory (impedance, pure tone audiogram)test, and musculoskeletal (bone marrow density [BMD] and range of motion [ROM]) measurements. Blood tests were conducted during intravenous line placement for MSC infusion, minimizing additional vascular access burden. To prevent occlusive complications following MSC administration, such as pulmonary embolism, enoxaparin (0.5 mg/kg) was administered subcutaneously before and after each infusion. The study was terminated prematurely due to death 10 months after initiation, 2 months after the 4th dose of MSCs. Treatment efficacy was evaluated by comparing three periods: the two years prior to treatment, the treatment period, and the posttreatment period, encompassing the natural progression of progeria. Adverse reactions were assessed via the WHO-UMC causality assessment system and the common terminology of criteria for adverse events (CTCAE). This study was registered with the Clinical Research Information Service (CRIS number: KCT0008336).

## 3. Results

### 3.1. Improvements in Body Composition, Bone Mineral Density, and Dentition

**Figure 1:**
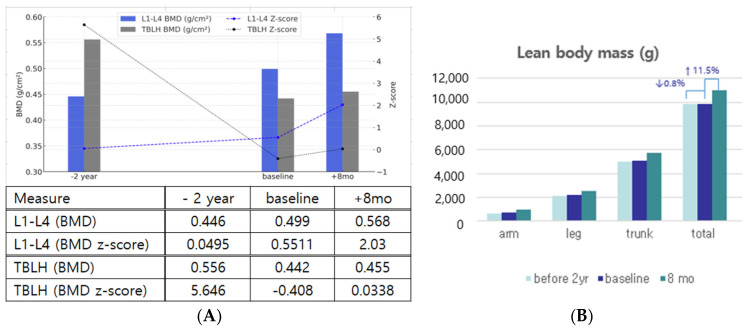
(**A**) Changes in bone mineral density and (**B**) lean body mass bone mineral density. TBLH—total body less head (z-score was assessed by age); BMD—bone mineral density; L1–L4—lumbar vertebrae 1–4 (z-score was adjusted by height-matched age). Lean body mass decreased (↓) 0.8% during pretreatment over 2 years, increased (↑) 11.5% after 8 months of treatment.

Following treatment, notable improvements in bone mineral density (BMD), lean body mass, and dentition status were observed. Body composition and bone mineral density were assessed using dual-energy X-ray absorptiometry (DXA). During the pretreatment period, a slight increase in L-spine BMD was observed, whereas TBLH BMD decreased. In contrast, both measures showed significant improvement after MSC therapy. When calculated as annual rates, L1–L4 BMD increased by 5.94% per year during the pretreatment period and accelerated to 20.74% per year after MSC therapy. In contrast, TBLH BMD showed a −10.25% annual decline before treatment but improved to a 4.41% annual increase following therapy. The L1-L4 BMD z-score also improved markedly from 0.0495 to 2.03, shifting from near average to well above the mean for height age-matched children, suggesting a positive effect of MSC therapy on bone metabolism (Figure 1A). Lean body mass also significantly increased by treatment. The annual rate of total body mass change shifted from −0.4% per year (−0.8% over 2 years) before treatment to +17.25% per year (+11.5% over 8 months) after MSC therapy, suggesting a potential anabolic effect (Figure 1B). Detailed data of changes in body composition and fat mass are provided in the Supplementary Materials.

All the primary teeth were retained before treatment. After MSC administration, the previously retained deciduous teeth exhibited mobility, rapid eruption of permanent teeth, and exfoliation of primary teeth following the first (#51, #52, #71), second (#81), and fourth (#61) MSC administrations.

### 3.2. Amelioration of Stiffness: Joints, Tympanic Membrane, and Arterial Flexibility

Treatment led to improvements in the stiffness of joint mobility, tympanic membrane, and arterial stiffness. The ROMs of the hip, knee, shoulder, and elbow joints increased slightly. However, the ROMs of the wrist and fingers did not improve (Figure S3). A decrease in tympanic membrane stiffness was observed via tympanometry, leading to improved hearing, as confirmed by pure tone audiometry (Figure S4A,B). BaPWV, an indicator of arterial stiffness, decreased by an average of 9.98% with treatment. Specifically, the velocity decreased from 1113 cm/s to 1011 cm/s on the right side and from 1228 cm/s to 1097 cm/s on the left side (Table 1). These findings indicate that the treatment effectively improved stiffness and overall physiological function.

### 3.3. Short-Term Improvement in Growth and Metabolic Aspects

Weight gain improved with MSC treatment, and after two months of treatment, the greatest improvement in all indicators except height (weight, IGF-1, IGFBP3, and HbA1c) was observed. A weight gain of 1 kg was observed during the 8-month treatment period, whereas only 0.5 kg was observed over the two-year pretreatment period. The change in the z-score during the post-treatment period was +0.75, whereas that during the pretreatment period was −1.13. The increase in the growth rate is less clear, but the rate of z-score deterioration has slowed. The IGF-1 level rose from 173.1 ng/mL (z score: 0.03) to 235.6 ng/mL (z score: 1.32), and the IGFBP3 level rose from 1786.5 ng/mL (z score: −1.6) to 2664.7 ng/mL (z score: 0.37) after 2 months of treatment. In terms of metabolism, glycemic control improved initially (glycated hemoglobin [HbA1c]: 5.8% → 5.5% in 2 months). However, by 6 months after the third MSC administration, these gains had diminished (Table 1). Metabolic abnormalities associated with steatotic liver disease (MASLD) were observed with abdominal ultrasound at baseline and did not progress during treatment (Table 1).

### 3.4. Reduction in Inflammatory Cytokines

**Figure 2:**
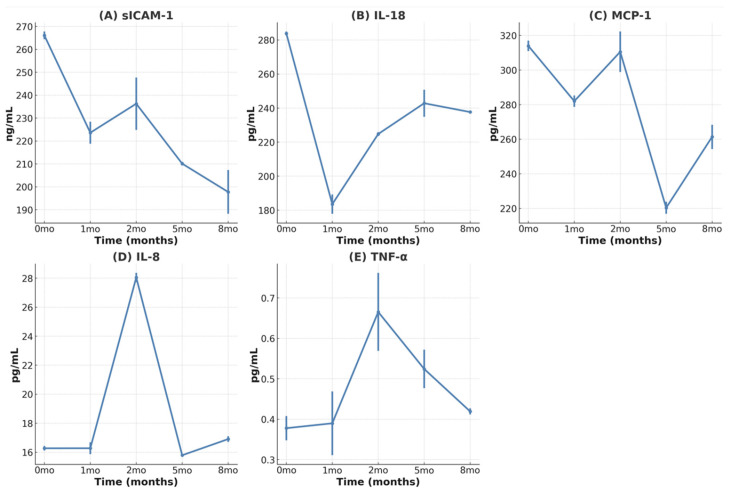
Changes in the levels of the following serum cytokines: (**A**) sICAM-1, (**B**) IL-18, (**C**) MCP-1, (**D**) IL-8, (**E**) TNF-α. sICAM-1—soluble intercellular adhesion molecule-1; IL-18—interleukin-18; MCP-1—monocyte chemoattractant protein-1; IL-8—interleukin-8; TNF-α—tumor necrosis factor-α; IL-1β—interleukin.

We evaluated the cytokines IL-18, MCP-1, sICAM-1, IL-8, TNF- α, and IL-1β. IL-1β was undetectable throughout the study. Cytokine changes in this study were interpreted based on intra-patient trends (Figure 2A–E). Systemic cytokine analysis showed a sustained decrease in sICAM-1 levels with repeated MSC administration, suggesting reduced vascular inflammation. IL-18, MCP-1, and sICAM-1 levels also decreased after the first dose, indicating an anti-inflammatory effect of MSC therapy. Increased IL-8 levels with repeated MSC treatment improve endothelial cell function by increasing endothelial nitric oxide synthase (eNOS) activity and reducing oxidative stress [26]. However, in the context of cytokine changes, we assume that the efficacy of MSCs decreases over time.

### 3.5. Lower Efficacy for Atherosclerosis and Cardiovascular Aspects

**Figure 3:**
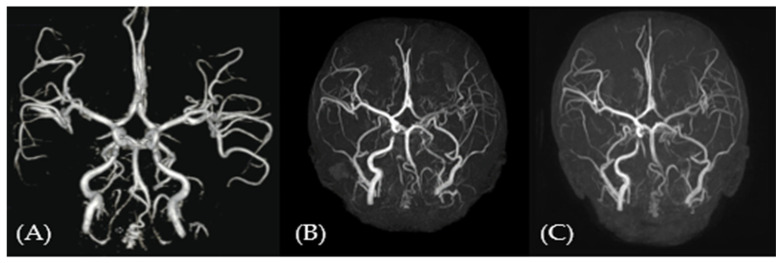
Changes in the cerebrovascular state before and after treatment were detected via brain magnetic resonance angiography (MRA). At diagnosis, brain MRA revealed vertebral artery (VA) occlusion and a tortuous internal carotid artery (ICA) with collateral vessels. (**A**) At baseline, diffuse hypoplastic changes with segmental total occlusion developed in the left ICA for two years before treatment, with sustained VA stenosis. (**B**) At 5 months after treatment, treatment did not completely halt vascular deterioration, and no significant progression was observed (**C**).

Despite treatment, atherosclerosis and cardiovascular deterioration did not stop or reverse. Cerebrovascular imaging demonstrated progressive changes over time. Two years before treatment, severe segmental stenosis of the bilateral vertebral arteries and mild stenosis of the bilateral ICA with collateral vessels were observed (Figure 3A). At baseline, vertebral artery stenosis had progressed, and hypoplastic changes with segmental total occlusion of the left ICA were identified (Figure 3B). Follow-up imaging showed some progression, especially in the left vertebral artery, but no new major vascular events or additional occlusions were observed (Figure 3C). These changes may reflect the natural course of cerebrovascular disease in HGPS.

On the other hand, atherosclerosis and cardiac dysfunction progression were evident. The atherosclerotic marker cIMT increased during treatment. At 5 months, the right mean cIMT increased from 0.47 mm to 0.61, exceeding the normal range (0.365–0.58 mm) [27]. Additionally, right maximum cIMT also increased from 0.60 to 0.80 mm. Progression of mild diastolic dysfunction was observed through tissue Doppler imaging (early diastolic velocity 7→5.89 cm/s normal > 8 cm/s, E/e 16.1→12.28 normal < 8). Systolic function, as measured by the ejection fraction and fractional shortening, was within the normal range but declined over time. No calcification was detected on TTE during treatment (Table 1).

### 3.6. Safety of MSC Treatment

Unfortunately, MSC therapy did not result in an extension of the patient’s lifespan. The study was terminated due to the patient’s death 10 months after treatment initiation at age 8 years and 7 months. The presumed cause of death was post-exercise arrhythmia, considered unlikely to be related to MSC treatment. The patient was dead on arrival with asystole on ECG, and no further evaluation was possible. An autopsy was not performed in accordance with the guardians’ wishes. Following a thorough investigation, both the Korea National Institute of Health (KNIH) and the Regenerative Medicine Acceleration Foundation reviewed the termination report and concluded that the death was not related to MSC therapy.

During MSC administration, the only adverse events that occurred within 24 h of administration were nausea, vomiting, and dizziness with the first dose. At the first dose, lorazepam was administered concurrently to relieve anxiety. Without lorazepam, the side effects do not appear repeatedly, so it looks less relevant to stem cell therapy. Hand weakness lasting for an hour and a half without headache at 6 days after the first dose might be associated with his basal vascular state, which is less likely with MSC therapy. Frequent epistaxis was thought to be due to the use of clopidogrel. Thirty-six days after the 4th MSC treatment, the patient reported intermittent, stress-induced chest pain lasting 1–2 min, which resolved completely. An immediate attempt to conduct additional evaluation was made, but scheduling delays due to patient refusal because of various circumstances resulted in unexpected death during daily activities. Other mild adverse reactions associated with infection were noted (Table S2).

## 4. Discussion

This study was systematically designed and is, to our knowledge, the first to administer a precise cell count of MSCs to a patient with progeria. Safety was ensured through precise quantification and repeated administration of MSCs, along with comprehensive monitoring.

Like lonafarnib [13], this study confirmed that MSC therapy also has positive effects on bone structure, vascular and joint stiffness, and low-frequency sensorineural hearing. Additionally, MSC therapy improved dentition and had anti-inflammatory effects, with short-term positive effects on growth and metabolism. The Lumbar spine, predominantly composed of metabolically active trabecular bone, showed significant improvement not only in BMD but also in the BMD z-score (0.0495 → 2.03), supporting the therapeutic effect of MSCs on bone metabolism. While the TBLH region, predominantly cortical bone with lower metabolic activity, showed smaller changes (BMD: 0.442 → 0.455 g/cm^2^, BMD z-score: −0.408 → 0.0338), it shifted from a declining trend to stabilization. These results suggest that MSC therapy may positively influence bone metabolism in HGPS, which is consistent with the known mechanism of impaired osteoblast differentiation and low bone turnover in this disease [28].

There were two prior cases of stem cell therapy in HGPS. A single use of adipose tissue-derived stromal vascular fractions containing MSCs improved the growth rate, weight gain, and IGF-1 levels [29]. Another patient treated with cord blood cells exhibited decreased PWV, increased height, weight gain, and increased ROM of the proximal joint [30]. These results were consistent with our study. However, unlike these studies or lonafarnib trial, our case did not show improvement in cIMT. This is likely due to the patient’s severe baseline vascular disease.

The patient rapidly progressed to vascular changes. He had bilateral vertebral artery stenosis at diagnosis, and additional ICA stenosis had developed over two years. Murtada et al. reported that the aortic phenotype worsened rapidly as the disease progressed toward the terminal stage in mouse models [31]. Despite active LDL cholesterol management using lipid-lowering agents to prevent stroke, atherosclerosis progression was evident on cIMT. This aligns with clinical trial data showing the combination therapy (lonafarnib + pravastatin + zoledronate) did not outperform lonafarnib alone [15]. The failure of lipid-lowering drugs to improve arteriosclerosis is thought to be attributed to a distinct mechanism specific to progeria. Progressive diastolic dysfunction, a characteristic finding in patients with progeria [32] was also observed in TTE. While the patient’s vascular deterioration progressed rapidly, it is unlikely that MSC therapy contributed to this outcome. This is supported by well-established cardioprotective properties of bone marrow-derived MSCs (BM-MSCs), including promotion of angiogenesis, reduction in fibrosis, and preservation of cardiac function [33]. Furthermore, MSCs have demonstrated therapeutic potential in acute myocardial infarction [34]. Considering that cerebrovascular deterioration begins at early age, we believe similar changes may have also occurred in the coronary arteries.

The clinically proven drug for cardiovascular manifestations in progeria is lonafarnib, the efficacy of which has been demonstrated in a mouse model through the use of progerinin [35] and antisense oligonucleotides (ASOs) [36]. These drugs effectively lower progerin levels. Unfortunately, progerin levels were not assessed in this study. Additionally, therapeutic approaches targeting endothelial cell dysfunction, such as Angiopoietin-2 inhibition and Verteporfin (a YAP/TAZ pathway inhibitor), have also been reported to improve vascular function. This study suggests that MSC therapy may improve vascular function, as evidenced by changes in baPWV and cytokine levels. Baseline measurements revealed an elevated baPWV in the patient, with a mean value of 1170 cm/s, which exceeds the 95th percentile for a healthy 12-year-old boy (1039 cm/s) [37]. Given that the PWV naturally increases with age and height, this value indicates a significant degree of arterial stiffness. After MSC treatment, the patient demonstrated a reduction in baPWV, suggesting an improvement in arterial stiffness and a positive therapeutic response. Additionally, serial reductions in sICAM-1, an endothelial activation and inflammation marker [38,39], suggest reduced vascular inflammation and potential atherosclerosis risk mitigation.

Due to rarity of HGPS, this study included a single subject, limiting the generalizability and making statistical analysis infeasible. Nonetheless, the findings provide valuable insights. Brain MRA follow-ups suggested that MSC therapy may alleviate deterioration but not halt or reverse structural changes. A low dose of MSCs (2.5 ×10^5^ cells/kg) was chosen to prioritize vascular safety considering the patient’s compromised vascular status, and history of TIA. Some effects of MSCs are short-lived and relatively insufficient. As safety was ensured throughout this study, dose escalation and earlier intervention-prior to irreversible changes- should be considered to maximize therapeutic benefits I future study. Additionally, progerin levels could not be measured in this study, which limits the ability to directly assess biological impact of MSC therapy on disease mechanism. To place these findings in context, we next review recent advances in stem cell–based therapies for HGPS, including clinical and experimental studies.

## 5. Literature Review

Recent stem cell research in HGPS spans mechanistic studies, disease modeling, and in vivo and clinical investigations, providing a foundation for novel therapeutic strategies (Table 2). Scaffidi and Misteli (2008) first demonstrated that progerin impairs MSC function by activating premature aging pathway [40]. Similarly, Rosengardten et al. (2011) reported the depletion of endogenous stem cell pools in HGPS, underscoring the importance of stem cell maintenance in disease pathology [19]. These findings suggest stem cell-based approaches, including replacement strategies. 

The advent of induced pluripotent stem cells(iPSCs) technology has significantly advanced HGPS research. Differentiation of iPSCs derived from HGPS fibroblasts into vascular smooth muscle cells and MSCs, offering valuable models of studying pathogenesis and paving the way for targeted therapies [41,42,43]. In an in vivo study, transplantation of muscle-derived stem/progenitor cells into progeroid mice notably extended both their healthspan and lifespan [44]. Clinical studies have also explored the efficacy of stem cell based therapies. Administration of allogeneic cord blood cells or adipose tissue derived stromal vascular fraction containing MSCs have shown improvements in growth and clinical parameters. However, these studies remain limted in duration and scope. 

Overall, recent advances in stem cell research including iPSC-based modeling, transplantation of stem cells, have deepened our understanding of HGPS and underscored the potential of regenerative medicine as a therapeutic avenue. A growing trend toward combination therapies that target multiple aspects of disease pathogenesis has emereged [45]. In particular, integrating stem cell therapy with agents that reduce progerin production, such as lonafarnib, may offer synergistic effects. Therefore, further studies should clarify underlying mechanisms, and optimize combination approaches to improve clinical outcomes. 

**Table 2:** Stem cell research on HGPS. hMSC- human Mesenchymal stem cells; HGPS—Hutchinson–Gilford progeria syndrome; SVF—stromal vascular fraction; iPSCs—induced pluripotent stem cells; MDSPCs—muscle-derived stem/progenitor cells.

Year Authors	Subjects	Methodology	Key Findings	Mechanism	Clinical Implications	Limitations/Future Directions
2008 [40] Scaffidi P et al.	hMSC	Expression of progerin in hMSCs; analysis of stem cell function	Discovered misregulation leading to premature aging	Progerin interferes with the function of hMSCs	Progerin activates Notch signaling pathway in hMSCs	Limited to in vitro study: in vivo confirmation needed
2011 [41] Zhang J, et al.	iPSC-derived cells	iPSC differentiation and analysis	Vascular smooth muscle and mesenchymal stem cell defects identified	iPSCs reveal specific cellular defects in HGPS	New targets for therapeutic intervention	Validation in patient samples required
2011 [42] Liu et al.	HGPS patient fibroblasts	Generation of iPSCs from HGPS fibroblasts; Differentiation of iPSCs	iPSCs from HGPS patients lack progerin expression but resume upon differentiation	Reprogramming suppresses progerin expression: differentiation resumes aging-associated phenotypes	iPSCs as a model for studying HGPS and drug screening	Limited to in vitro model: in vivo validation needed
2011 [19] Rosengardten Y et al.	HGPS mouse model	Analysis of stem cell populations and wound healing capacity	HGPS mutation causes adult stem cell depletion and impaired wound healing	Progerin accumulation leads to stem cell exhaustion	Stem cell therapies may be beneficial for HGPS patients	Limited to mouse model: human studies needed
2012 [44] Lavasani M et al.	Progeroid mice	Intraperitoneal injection of young wild-type MDSPCs	Extended lifespan and healthspan of progeroid mice	Secretion of factors by MDSPCs that improve tissue function	Stem cell transplantation as potential therapy for progeria	Limited to animal model: human studies needed
2015 [43] Lo Cicero A, Nissan X	iPSCs	iPSC modeling of HGPS	Improved understanding of disease mechanisms	iPSCs recreate HGPS cellular environment for study	Improved drug screening platform	Translation to in vivo models needed
2020 [29] Park J et al.	HGPS patient	adipose SVF containing MSC	Increased height, weight and IGF-1	anti-inflammatory effects via paracrine signaling	Proposal for the potential treatment of inflammaging-related diseases	Single case study: larger trials needed
2021 [30] Suh YS, et al.	HGPS patient	Cord blood stem cell infusion +sirolimus	Improved growth, reduced PWV, slowed IMT progression	Cord blood stem cells may provide trophic support and replace damaged cells	Potential noninvasive treatment for HGPS	Single case study: larger trials needed

## 6. Conclusions

Herein, we report the case of a patient with HGPS who received MSC therapy. Our findings suggest that MSC therapy for HGPS is safe and may have potential benefits, including increased bone mineral density, reduced joint stiffness and arterial stiffness, and anti-inflammatory effects. Given that a favorable safety profile was observed in this case, further studies with optimized dosing strategies and earlier intervention before significant vascular deterioration are warranted. Additionally, a personalized protocol with long-term monitoring should be developed to optimize therapeutic outcomes. Since MSC therapy has limited effects on cardiovascular disease, it may be more effective as an adjuvant therapy to lonafarnib than as monotherapy. Furthermore, beyond HGPS, stem cell therapy warrants further investigation for its potential role in aging related conditions, cardiovascular disease prevention, and the treatment of rare diseases.

## Figures and Tables

**Table 1:** Changes in growth, metabolic, and cardiac indicators over time.

		−2 Years	Baseline	2 Months	5 Months	8 Months
			Before Treatment	1 Month After 2nd MSC	3 Months After 3rd MSC	6 Months After 3rd MSC
Growth	Height (cm) z-score for height	93.2 −4.65	101.2 −5.41	102.1 −5.41	102.2 −5.46	103 −5.73
	Weight (kg) z-score for weight	11.6 −5.56	12.1 −6.89	12.6 −6.48	12.7 −6.3	13 −6.14
	ALP (U/L) (ref: 120–344)	178	207	218	166	207
	IGF-1 (ng/mL) (ref: 90.6–268.8)	145.5	173.1	235.6	118	104.4
	z-score for IGF-1	−0.15	0.03	1.32	−1.24	−1.49
	IGFBP3 (ng/mL) (ref: 1620–3490)	1446	1786	2664	2608	2147
	z-score for IGFBP3	−1.96	−1.6	0.37	−0.04	−0.96
Metabolic	HbA1c (%)		5.8	5.5	5.9	5.8
	AST/ALT (U/L)	31/22	34/35	38/38	31/41	34/45
	Cholesterol (mg/dL)	219	180	165	149	115
Cardiac	CK-MB (normal 0–5 ng/mL)	4.2	3.9	2.7	1.9	3.2
	D-dimer (normal 0–0.5 μg/mL)		1.67	0.40	0.37	0.28
	BP (systolic/diastolic)	99/69	100/47	104/51	100/52	117/60
	BaPWV (cm/s) rt/lt		1113/1228		1011/1097	
	cIMT mean (mm) rt/lt	0.43/0.36	0.47/0.46		0.61/0.51	
	max (mm) rt/lt	0.60/0.52	0.60/0.68		0.80/0.68	
	TTE-TDI e’ (cm/s (z-score)) E/e (normal < 8)		7 (−4.04)		8 (−3.53) 16.1	5.89 (−4.6) 12.28
	EF (normal 50–70%)	64.36	70		62.7	56.2
	FS (normal 25–45%)	34.38	40		33.18	28.48

Weight, height, IGF-1, and IGFBP3 increased in the short term and Hba1c decreased in the short term. IGF-1—insulin-like growth factor-1; IGFBP3—insulin-like growth factor-binding protein. Hba1c—glycated hemoglobin; BaPWV—brachial ankle pulse wave velocity; cIMT—carotid intima media thickness; TTE—transthoracic echocardiogram; TDI—tissue Doppler imaging; e’—early diastolic velocity; E/e—mitral inflow E-wave-to-early diastolic velocity ratio. EF—ejection fraction; FS—fractional shortening. Bold text indicates deterioration or out of the normal range.

## Data Availability

The original contributions presented in the study are included in the article/Supplementary Materials, further inquiries can be directed to the corresponding authors.

## Supplementary Materials

The following supporting information can be downloaded at: https://www.mdpi.com/article/10.3390/children12040523/s1. Figure S1 Changes in body composition; Figure S2. Transthoracic echocardiography (TTE) image of patient; Figure S3. Change of range of motion from baseline; Figure S4. Change of Audiological Evaluation; Table S1. Changes in body composition; Table S2. Lists of adverse reactions.
