# Supplementary material for: Mesenchymal Stem Cell Therapy for Hutchinson–Gilford Progeria: Improvements in Arterial Stiffness and Bone Mineral Density in a Single Case
Source: Children (Basel). 2025 Apr 18;12(4):523. doi: 10.3390/children12040523 (PMC12025413; doi:10.3390/children12040523)
Supplement: Supplementary file 1 [file children-12-00523-s001.zip › children-3500174-supplementary.pdf]

children

MDPI

Supplementary Table S1. Changes in body composition

		-2 year	baseline	+8months
Bone mineral content		318	467	502
lean	arm	679	748	944
	change rate	+9.22%	+26.1%	
	leg	2104	2207	2532
	change rate	+4.67%	+14.72%	
	trunk	4980	5083	5736
	change rate	+2.02%	+12.83%	
	total	9870	9862	11000
	change rate	-0.8%	+11.5%	
Fat		966	1909	1852

Suppelementary Figure S1. Changes in body composition.

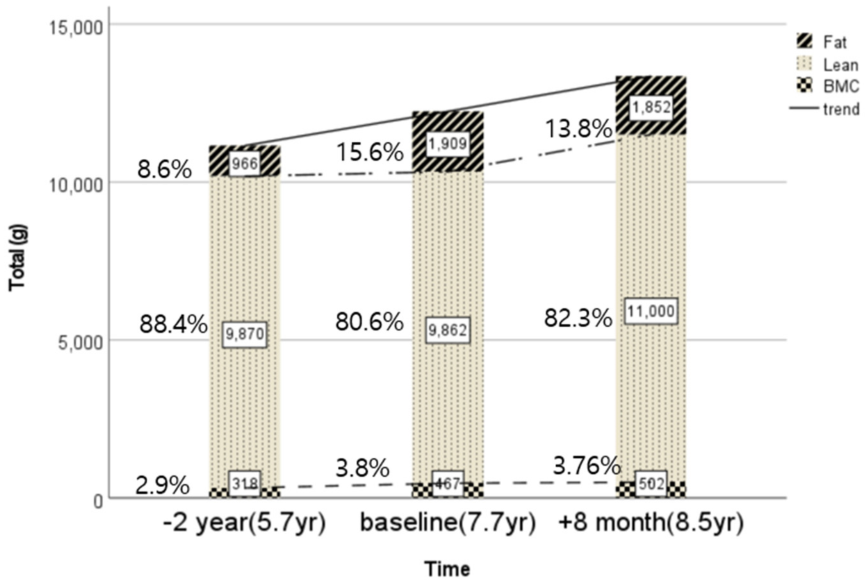

Supplementary Figure S2. Transthoracic echocardiography (TTE) image of patient.

(A) **(B)** **(C)**

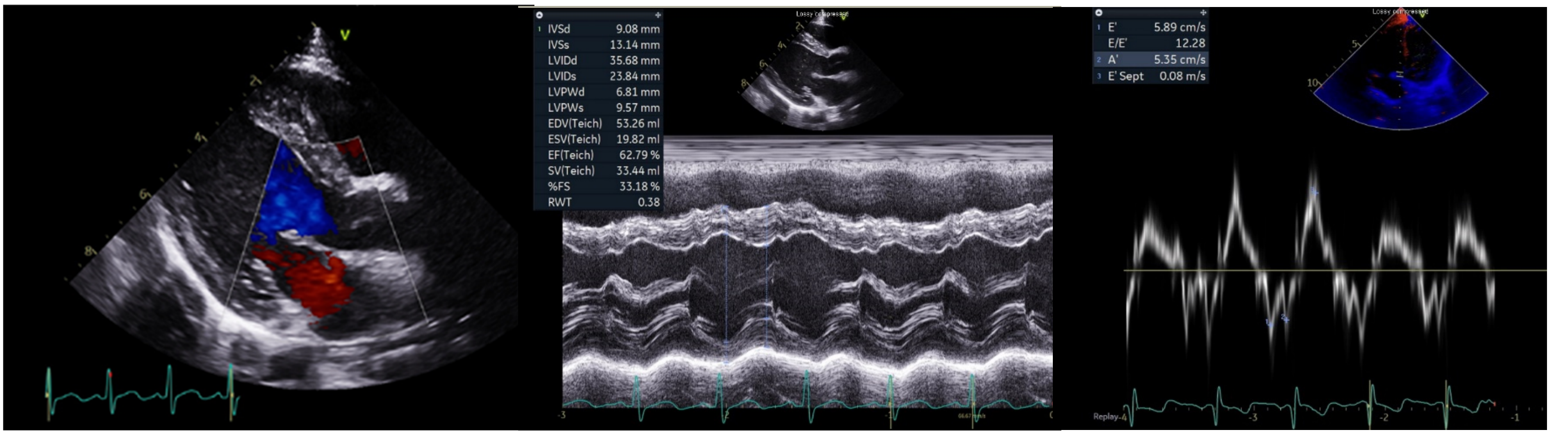

(A) Color Doppler and (B) M-mode images at 5 months after treatment. (C) Tissue Doppler imaging (TDI) of the septal annulus at 8 months

Supplementary Figure S3. Change of range of motion from baseline

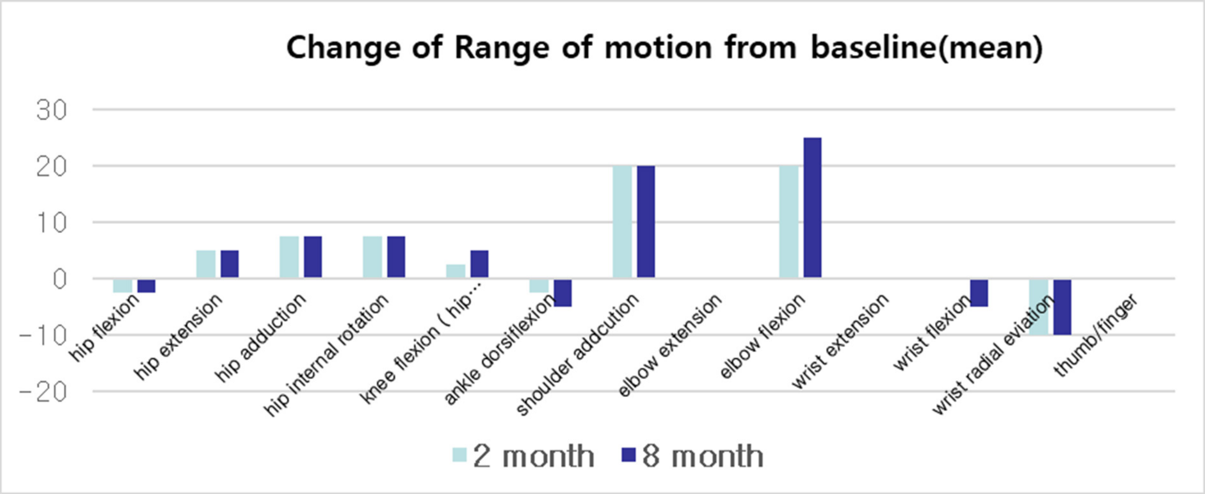

Supplementary Figure S4. Change of Audiological Evaluation

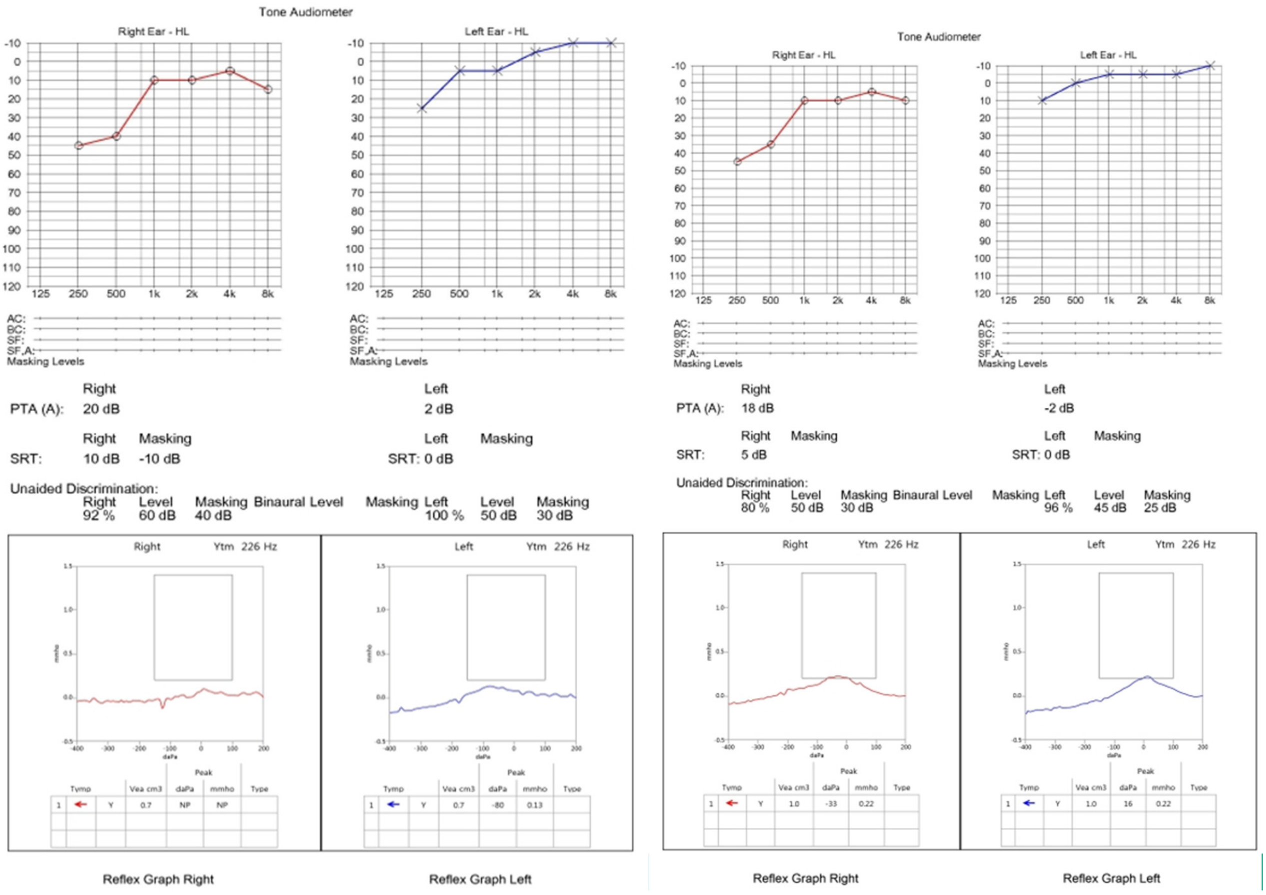

(A) baseline (B) after 8 month

Suplementary Table S2. Lists of adverse reactions.

	Symptoms	Time	Intervention	Outcome	Severity	Causality	Concomitant
**Gastro intestinal**	Nausea/ Vomiting	3.5 hr after 1st dose	IV hydration	Resolved completely	Mild (grade 1)	Unlikely	After 1st dose d/t ativan
**Neurological**	Dizziness	3.5 hr after 1st dose	IV hydration	Resolved completely	Mild (grade 1)	Unlikely	After 1st dose d/t ativan
	**Hand weakness**	Duration-1.5 hr<br>6 days after 1st dose	observation	Resolved completely	Moderate (grade2)	Unlikely	d/t progeria
**Constitutional**	Fever	1day before 3rd dose<br>103 day after 3rd dose	antipyretics	Resolved completely	Mild (grade 1)	Unlikely	d/t viral infection, AOM
**Hematological**	Epistaxis	1day before 3rd dose<br>97 days after 3rd dose<br>98 days after 3rd dose	observation	Resolved completely	Mild (grade 1)	Unlikely	d/t Plavix
**Respiratory**	Cough	26 days after 3rd dose ~ approximately 2 month	PO med	Resolved completely	Mild (grade 1)	Unlikely	d/t infection
	Rhinorrhea	26 days after 3rd dose approximately 2 month	PO med	Resolved completely	Mild (grade 1)	Unlikely	
	Otitis media	26 days after 3rd dose ~ approximately 2 month	PO antibiotics, NSAIDS	Resolved completely	Mild (grade 1)	Unlikely	
**Skin**	Furuncle	3 month after 3rd dose	Mupirocin, PO antibiotics	Resolved	Mild (grade 1)	Unlikely	d/t progeria
**Cardiac**	Chest discomfort	36 days after 4th dose<br>39 days after 4th dose<br>40 days after 4th dose	Further evaluation recommended, but refused	Resolved completely	moderate (grade 2)	Unlikely	d/t progeria
	Death	60 days after 4th dose	-	-	Severe (grade 5)	Unlikely	d/t progeria
